# The effects of layer-wise relevance propagation-based feature selection for EEG classification: a comparative study on multiple datasets

**DOI:** 10.3389/fnhum.2023.1205881

**Published:** 2023-06-05

**Authors:** Hyeonyeong Nam, Jun-Mo Kim, WooHyeok Choi, Soyeon Bak, Tae-Eui Kam

**Affiliations:** Department of Artificial Intelligence, Korea University, Seoul, Republic of Korea

**Keywords:** brain-computer interface, feature selection, layer-wise relevance propagation, motor imagery classification, electroencephalography, analysis

## Abstract

**Introduction:**

The brain-computer interface (BCI) allows individuals to control external devices using their neural signals. One popular BCI paradigm is motor imagery (MI), which involves imagining movements to induce neural signals that can be decoded to control devices according to the user's intention. Electroencephalography (EEG) is frequently used for acquiring neural signals from the brain in the fields of MI-BCI due to its non-invasiveness and high temporal resolution. However, EEG signals can be affected by noise and artifacts, and patterns of EEG signals vary across different subjects. Therefore, selecting the most informative features is one of the essential processes to enhance classification performance in MI-BCI.

**Methods:**

In this study, we design a layer-wise relevance propagation (LRP)-based feature selection method which can be easily integrated into deep learning (DL)-based models. We assess its effectiveness for reliable class-discriminative EEG feature selection on two different publicly available EEG datasets with various DL-based backbone models in the subject-dependent scenario.

**Results and discussion:**

The results show that LRP-based feature selection enhances the performance for MI classification on both datasets for all DL-based backbone models. Based on our analysis, we believe that it can broad its capability to different research domains.

## 1. Introduction

Brain-computer interface (BCI) enables individuals to connect with their surroundings by establishing communication channels between the brain and external devices using their neural signals (McFarland and Krusienski, 2012). The BCI systems have been developed for a variety of applications including communications, healthcare, military services, and rehabilitative technologies (Daly and Wolpaw, 2008; Mcfarland and Wolpaw, 2010; Van Erp et al., 2012; Biasiucci et al., 2018; Belkacem et al., 2020). One popular paradigm in BCI research is motor imagery (MI), which involves imagining specific movements without actually performing them such as movements of arms or other body parts to generate neural signals that can be decoded to control the devices (Jeannerod, 1994; Zhang et al., 2021b). To perform MI tasks, participants are usually guided by predefined conditions and time intervals, with visual or auditory cues provided throughout the task to help them imagine the movements during specific time periods (Lotte et al., 2018). It is known that MI produces identical neural responses on the motor and sensorimotor regions (Jeannerod, 1995; Lotze and Halsband, 2006; Vyas et al., 2018). This capability that measures the human intention of specific actions enables to transfer desired signals into BCI systems, which will also lead to a variety of future applications. Electroencephalography (EEG) is a common measurement used in the MI-BCI field to obtain the electrical signals of the brain through electrodes placed on the scalp (Blankertz et al., 2010; Millán et al., 2010). EEG signals have the advantage of the non-invasive nature and high temporal resolution (Collinger et al., 2014; Lotte et al., 2018), and also capture motion-related information across the spatial, temporal, and spectral domains (Dai et al., 2020). The research on EEG-based motor imagery classification contributes to unraveling the neural mechanism (Pfurtscheller and Neuper, 2001) and paves the way to develop more sophisticated systems in areas such as real-time BCI controls, stroke rehabilitation, and assistive technologies for paralyzed individuals (Ang et al., 2011; Leeb et al., 2011; Pichiorri et al., 2015; Shin et al., 2022; Forenzo et al., 2023).

Deep learning-based approaches have been a growing trend in EEG-based motor imagery classification, especially adopting convolutional neural networks (CNN) (Tabar and Halici, 2016; Schirrmeister et al., 2017; Lawhern et al., 2018; Li et al., 2019; Zhang et al., 2019; Altuwaijri and Muhammad, 2022; Chen et al., 2022; Huang et al., 2022; Lee et al., 2022; An et al., 2023; Wang et al., 2023b), as it takes advantages to learn more robust features that are not restricted to specific feature domains (Hertel et al., 2015). The CNN-based architectures can capture the spatial, temporal, and spectral features of EEG signals through several convolutional blocks, in which the general feature representation is essential when training neural networks. Specifically, Schirrmeister et al. (2017) have utilized CNN variants such as DeepConvNet and ShallowConvNet to decode EEG signals for MI classification through general feature representations. Lawhern et al. (2018) have proposed EEGNet, another generalized deep learning architecture for EEG-based applications using separable convolutions and depthwise convolutions. Li et al. (2019) and Zhang et al. (2019) have also proposed hybrid neural networks with CNN variants and other deep learning frameworks for MI classification. Even in recent studies, CNN-based methods still remain dominant in MI-BCI (Altuwaijri and Muhammad, 2022; Chen et al., 2022; Huang et al., 2022; Lee et al., 2022; An et al., 2023; Wang et al., 2023b).

However, the use of all extracted EEG features from the well-known models does not always ensure high performance (Chatterjee et al., 2019). EEG has a low signal-to-noise ratio and high intra-variability of responses within subjects (Rakotomamonjy et al., 2005), which may result in classification errors. Therefore, selecting class-discriminative features from the extracted features is essential to improve classification performance (Luo et al., 2016). Selecting the class-discriminative features could also remove irrelevant or redundant features, resulting in more robust classifiers. Several studies have investigated various feature selection strategies for EEG-based MI-BCI in spatial, spectral, and temporal domains. Specifically, Zhang et al. (2021a) have extracted time-frequency features through wavelet transformation and selected crucial EEG channels via squeeze-and-excitation blocks. In addition, recent studies focusing on EEG feature selection have been actively considering various combinations of the spatial, temporal, and spectral domains through a range of approaches (Abbas and Khan, 2018; Liu et al., 2022; Sadiq et al., 2022; Tang et al., 2022; Luo, 2023; Meng et al., 2023).

Our recent work (Nam et al., 2023) has also conducted feature selection for EEG-based motor imagery classification based on Layer-wise relevance propagation (LRP) (Bach et al., 2015). LRP is a method designed to analyze and understand how a model processes information or makes decisions by providing the importance of input features through the decomposition of the prediction output backward (Bach et al., 2015). There has been a growing interest in utilizing LRP in BCI, where Sturm et al. (2016) and Bang et al. (2021) found neurophysiologically significant patterns with LRP-based generated heatmaps. This is because the interpretation of neural networks through LRP has proven consistent with corresponding domain knowledge in various fields (Lomazzi et al., 2023; Majstorović et al., 2023; Wang et al., 2023a). In addition, Nagarajan et al. (2022) also performed channel selection using relevance scores for each channel to improve the performance of MI classification. Capitalizing on these advantages, we have explored the potential of LRP for effective EEG feature selection on spatial, temporal, and spectral domains for motor imagery classification in our recent study. However, the prior work has only explored the feasibility of LRP-based feature selection using a single backbone network and a single dataset.

In this study, we take a further step and demonstrate the effectiveness of our LRP-based feature selection method on various backbone networks and datasets, as well as further comprehensive analysis that can identify performance improvement according to the feature selection. Given the transparency and the high level of interpretability of the LRP, we show that employing the LRP-based feature selection will lead to not only enhanced performance but also an intuitive and explainable feature selection process. Moreover, our study highlights the potential of applying our LRP-based feature selection approach to various cognitive and personal value EEG processes beyond motor imagery classification, extending its applicability to emotion recognition, attention monitoring, and BCI for meditation or relaxation. The development of more accurate and efficient methods for these processes could contribute to improving the quality of life for individuals through personalized technology solutions tailored to their specific needs and preferences. Thus we believe that the insights gained from our investigation emphasizes the broad impact of our research findings across different research domains.

## 2. Materials and methods

### 2.1. Dataset and preprocessing

To analyze the effectiveness of the LRP-based feature selection, we have evaluated our proposed method on two publicly available MI-EEG datasets, i.e., BCI Competition IV-2a dataset (Brunner et al., 2008) and KU-MI dataset (Lee et al., 2019). A brief summary of each dataset is described in the Table 1.

**Table 1:** A brief description of the BCI Competition IV-2a (Brunner et al., 2008) and KU-MI (Lee et al., 2019) datasets.

**Dataset**	**# Subject**	**# Class**	**# Session**	**# Trial per session**	**# Electrode**	**Sampling rate (Hz)**	**MI duration (s)**
BCI Competition IV-2a	9	4	2	288	22	250	4
KU-MI	54	2	2	200	62	1,000	4

The BCI Competition IV-2a dataset (Brunner et al., 2008) contains EEG recordings from nine healthy subjects performing four different motor imagery tasks as part of a cue-based MI-BCI paradigm. The tasks involve imagining the movements of the left hand, right hand, feet, and tongue, respectively. A total of two sessions on separate days were conducted for each subject, and each session includes six runs with short intervals in between. A single run consists of 48 trials (i.e., 12 trials for each task), resulting a total of 288 trials per session. The EEG recordings were generated using 22 Ag/AgCl electrodes according to the international 10–20 system (Homan et al., 1987). The signals contain 4-s MI that were recorded in a monopolar configuration, with the reference electrode placed on the left mastoid and the ground electrode on the right mastoid. The data were sampled with 250 Hz and bandpass-filtered between 0.5 and 100 Hz.

The KU-MI dataset (Lee et al., 2019) comprises of a large number of EEG recordings across 54 healthy subjects, where each subject participated in two different sessions. The dataset consists of two classes, which are 4-s left or right hand MI, where each session comprises 200 trials, with 100 trials for the left hand and 100 trials for the right hand. The EEG signals were captured using 62 Ag/AgCl electrodes with a sampling rate of 1,000 Hz according to the international 10–20 system.

For each dataset, we used 4.5-s EEG signals ranging from 0.5 s before the start cue to 4 s after the start cue. We then applied band-pass filtering to the signals, keeping the frequencies in the range of 0.5–40 Hz. This can be attributed to the fact that the most useful information from motor imagery signals can be found in the mu and beta frequency bands of the EEG, rather than in higher frequency bands (Dornhege et al., 2007; Kirar and Agrawal, 2018).

We also have standardized the continuous EEG data via exponential moving standardization to filter out noisy fluctuations. For the KU-MI dataset, we downsampled the EEG signals from 1000Hz to 250Hz. These preprocessing steps resulted in a two-dimensional EEG data format, with the number of electrodes (or channels) and time points represented as the dimensions (e.g., 22 × 1,125 for the BCI Competition IV-2a dataset and 62 × 1,125 for the KU-MI dataset). These measures were taken to ensure that both datasets were comparable and could be used for fair experimentation.

### 2.2. Proposed method

#### 2.2.1. Spatio-spectral-temporal feature extraction

**Figure 1:**
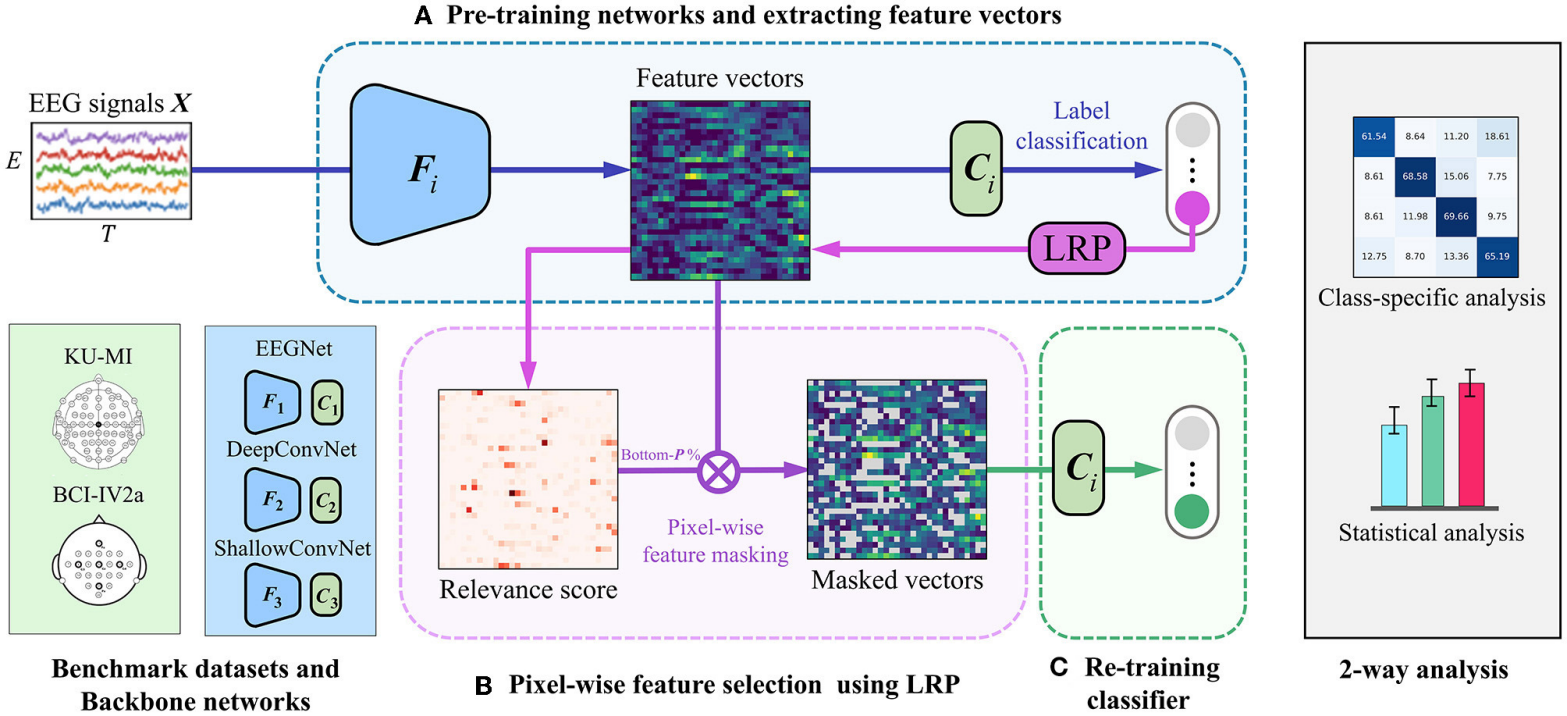
An overview of our proposed LRP-based feature selection framework is presented. To validate the framework's generalized performance, we evaluated it using three backbone networks on different datasets. The backbone networks include EEGNet (Lawhern et al., 2018), DeepConvNet (Schirrmeister et al., 2017), and ShallowConvNet (Schirrmeister et al., 2017). We tested the framework on two publicly available motor imagery datasets: the KU-MI dataset (Lee et al., 2019) and the BCI Competition IV-2a dataset (Brunner et al., 2008). Additionally, we conducted class-specific and statistical analyses afterward. The framework consists of three main steps: **(A)** a backbone network extracts EEG features and generates initial predictions, **(B)** feature-wise importance scores are calculated using the LRP method, and low-importance pixels in the feature vectors are masked, and **(C)** the classifier *C* is retrained using the selected non-masked features.

Figure 1 illustrates the overview of our LRP-based feature selection framework. It involves three well-known CNN-based backbone networks, EEGNet (Lawhern et al., 2018), DeepConvNet (Schirrmeister et al., 2017), and ShallowConvNet (Schirrmeister et al., 2017). They all can be described as a general model across EEG-based BCI paradigms, and have been shown good performance in MI classification tasks (Zhu et al., 2022). Each network is composed of a feature extractor (*F*) and a classifier (*C*), respectively. In each backbone network, the feature extractor *F* is trained with a series of spatial and temporal convolutional layers to learn spatio-spectral-temporal feature representations of the input EEG signals from various perspectives. The classifier *C*, which consists of a fully-connected layer, produces predicted class labels with their probability values by taking the extracted features from *F* as input.

**Figure 2:**
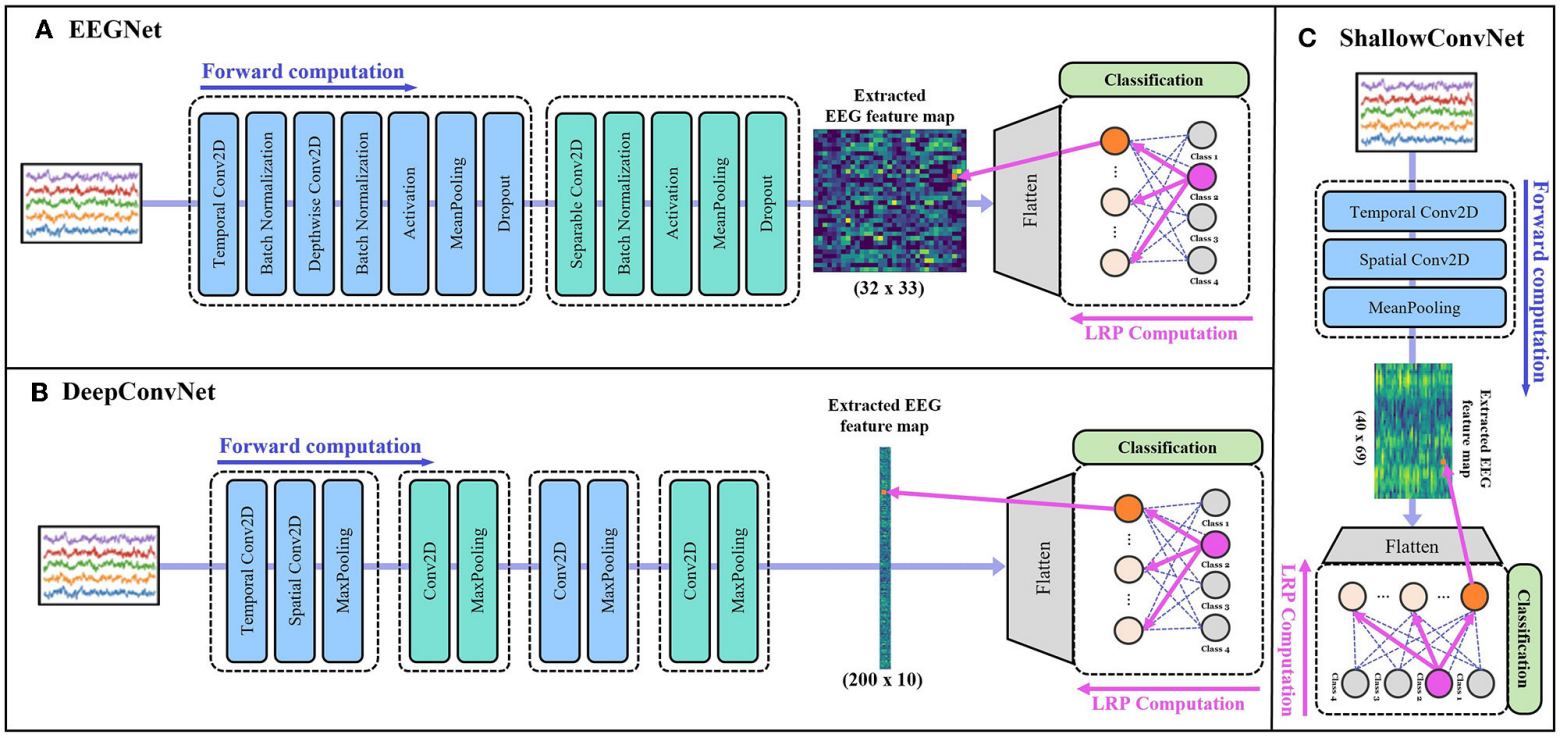
The structures of backbone networks: **(A)** EEGNet (Lawhern et al., 2018), **(B)** DeepConvNet (Schirrmeister et al., 2017), and **(C)** ShallowConvNet (Schirrmeister et al., 2017), respectively. Within each section, the feature extraction process of the corresponding model is shown up to the extracted EEG feature map, followed by the classification process and a schematic illustration of the LRP procedure for a specific input value until the feature map, that was generated by each backbone network.

More specifically, DeepConvNet (Schirrmeister et al., 2017) consists of four convolution blocks with max-pooling operations and a dense layer for classification. The first convolution block is specially designed to handle raw EEG signals, which enables to extract features from various perspectives. ShallowConvNet (Schirrmeister et al., 2017) comprises two convolution blocks with average pooling operations and a dense layer for classification. EEGNet (Lawhern et al., 2018) is another generalized deep learning architecture for EEG applications that employ depthwise and separable convolution blocks. The architecture is composed of three convolution blocks and a classification layer. Figure 2 shows the overall architecture of each backbone network.

#### 2.2.2. LRP-based feature selection

We measure the important scores for each extracted feature based on the layer-wise relevance propagation (LRP) method (Bach et al., 2015). The LRP is a well-known and commonly used framework where it helps to explain a neural network's decision-making process by decomposing down into relevance scores attributed to each neuron and enables highlighting important neurons in each layer toward a specific prediction (Bach et al., 2015; Montavon et al., 2017).

In a deep neural network, each neuron can be simply described as the following equation when computing the feed-forward:


$$\begin{array}{l} x_{j}^{\left(l+1\right)}=g\left(\underset{i}{\sum }x_{i}^{\left(l\right)}w_{ij}^{\left(l,l+1\right)}+b_{j}^{\left(l+1\right)}\right), \end{array}$$

where $x_{j}^{\left(l+1\right)}$ indicates *j*-th neuron at the layer *l*+1, and $\underset{i}{\sum }$ runs over all the neurons at the previous layers connected to the *j*-th neuron (Binder et al., 2016). The *g*(·) represents an activation function, and the parameters $w_{ij}^{\left(l,l+1\right)}$ and $b_{j}^{\left(l+1\right)}$ correspond to the weights and bias of the neuron, respectively. The final output of the neural network can be denoted by *f*(*x*) and this will become the very first relevance, which is the starting point for the LRP (Binder et al., 2016). The LRP method re-distributes the relevance *f*(*x*) into the relevance of the neuron in the preceding layer with the following rule, satisfying the desired conservation property $\Sigma_{p}R_{p}^{\left(1\right)}=f\left(x\right)$:


$$\begin{array}{l} R_{i}^{\left(l\right)}=\underset{j}{\sum }\frac{z_{ij}}{\underset{i^{\prime }}{\sum }z_{i^{\prime }j}+\epsilon \text{sign}\left(\underset{i^{\prime }}{\sum },z_{i^{\prime }j}\right)}R_{j}^{\left(l+1\right)}\;\left(z_{ij}=x_{i}^{\left(l\right)}w_{ij}^{\left(l,l+1\right)}\right). \end{array}$$

$R_{i}^{\left(l\right)}$ indicates the relevance of the neuron *i* at the layer *l*, where $\underset{j}{\sum }$ runs over all the neurons at preceding layers that are connected to the neuron *i* (Binder et al., 2016). The given rule is one of the variations of the LRP rule with a stabilizing factor $\epsilon \text{sign}\left(\underset{i^{\prime }}{\sum },z_{i^{\prime }j}\right)$ added to the denominator of the naive LRP rule, which prevents the denominator from becoming zero (Binder et al., 2016). Note that when the method reaches to the last layer, it generates a relevance map (heatmap) that visualizes the importance of each feature.

By applying this rule, we can compute the relevance scores (or importance scores) of each feature in the extracted feature map determining the degree of influence that each feature has on the decisions made by the model. Based on the importance score, we mask the irrelevant features that have low important scores. Figure 2 illustrates the overall process of feature extraction and how the LRP works from a certain output value until the feature map, that was generated by each backbone network.

#### 2.2.3. Classifier retraining

In order to improve the performance and accuracy of our model, we employ a strategy where we re-train the classifier *C* that we have used previously, using the masked feature vectors obtained during the training process. Retraining the classifier with masked feature vectors allows it to effectively capitalize on the most critical features while disregarding less relevant ones, ultimately contributing to better classification performance. To accomplish this, we intentionally freeze the feature extractor *F* within the backbone network architecture. By doing so, we prevent any updates or modifications to the learned features and solely focus on refining the classifier's ability to make accurate predictions based on the existing feature representations.

## 3. Results

### 3.1. Experimental settings

The backbone networks utilized in the experiment include EEGNet (Lawhern et al., 2018), DeepConvNet (Schirrmeister et al., 2017), and ShallowConvNet (Schirrmeister et al., 2017). These networks were selected for their extensive use in the BCI field as general-purpose architectures, where comparative evaluation of their performance have been already conducted in existing works to assess their effectiveness (Zhu et al., 2022). For evaluation, we followed the original structure of these networks as outlined in their respective papers, including pooling modes, activation functions, and kernel sizes, adhering to the specific recommendations provided by the authors. For instance, in EEGNet, we adjusted the kernel size of the temporal convolutional layer to half of the input data's sampling rate (1 × 125), and the kernel size of the depthwise convolutional layer to match the number of channels in our dataset, with sizes of (22 × 1) for the BCI Competition IV-2a dataset and (62 × 1) for the KU-MI dataset, respectively.

In the backbone network training process, we applied different hyper-parameter settings to have as the best performance as we can on each dataset, respectively. Specifically, for the BCI Competition IV-2a dataset, we adopted the Adam optimizer with a learning rate of 0.002 and the cosine annealing scheduler (Loshchilov and Hutter, 2016), and set the batch size of 72. For the KU-Mi dataset, we applied the RMSProp optimizer with a learning rate of 0.001 and the exponential scheduler (Li and Arora, 2019) with a batch size of 5.

For generalization in the classifier retraining process, we have given the networks the same hyper-parameter settings regardless of the datasets, by adopting the Adam optimizer with the learning rate of 0.002 and the cosine annealing scheduler. We set the batch size to be one-fourth of the total numbers of samples on each dataset, i.e., 72 and 50 for BCI Competition IV-2a and KU-MI datasets, respectively. In the LRP-based feature selection process, we masked 10% of the extracted features from each backbone network, respectively.

### 3.2. Performance evaluation

To access the efficacy of our feature selection technique, we have compared three different CNN-based backbone networks and our proposed feature selection method applied to each network on two publicly available datasets. As previously mentioned, the three backbone networks EEGNet (Lawhern et al., 2018), DeepConvNet (Schirrmeister et al., 2017), and ShallowConvNet (Schirrmeister et al., 2017) are used for the evaluation. The datasets we have used are the BCI Competition IV-2a dataset (Brunner et al., 2008) and KU-MI dataset (Lee et al., 2019), both of which consist of two sessions. The first session of each dataset was utilized as the training set, while the second session from each dataset served as the test set for analysis, following the subject-dependent scenario. To ensure the reliability of our results, we performed all experiments with five random seeds and measured the average accuracy of the seeds for each method.

Table 2 shows the performance comparison on the BCI Competition IV-2a dataset. The comparison results show that our proposed LRP-based feature selection method contributes to improving the performances when applied to all backbone networks. Specifically, our proposed method achieved a performance improvement of 0.86% [*p* < 0.05, Wilcoxon's signed-rank test (Wilcoxon, 1992)] in EEGNet, 0.37% (*p* = 0.1) in DeepConvNet, and 1.45% (*p* < 0.01) in ShallowConvNet, respectively, in terms of mean accuracy across all subjects.

**Table 2:** The performance comparison table of methods with and without the proposed LRP-based feature selection on the BCI Competition IV-2a dataset for different backbone networks.

**Comparison methods**		**Subjects**									**Mean**
		**S01**	**S02**	**S03**	**S04**	**S05**	**S06**	**S07**	**S08**	**S09**	
EEGNet	Backbone	77.29 ± 3.03	**59.17** **±2.39**	88.47 ± 2.92	65.42 ± 1.88	71.18 ± 2.29	**60.21** **±2.02**	72.71 ± 1.31	77.22 ± 1.88	76.32 ± 4.59	72.00 ± 0.51
	Proposed	**78.20** **±3.38**	58.82 ± 2.67	**89.79** **±2.56**	**66.18** **±2.76**	**72.57** **±2.70**	59.93 ± 3.18	**73.19** **±2.98**	**78.89** **±2.78**	**78.13** **±4.34**	**72.86** ±0.71^*^
DeepConvNet	Backbone	**76.46** **±1.38**	46.87 ± 2.04	87.92 ± 1.46	67.78 ± 3.15	74.38 ± 1.76	56.53 ± 2.10	74.58 ± 2.11	77.22 ± 1.94	**82.64** **±1.61**	71.60 ± 1.09
	Proposed	76.25 ± 1.83	**47.50** **±1.60**	**87.99** **±2.25**	**68.40** **±2.75**	**75.28** **±1.13**	**57.22** **±2.94**	**74.58** **±0.90**	**78.12** **±1.85**	82.36 ± 1.37	**71.97** **±0.79**
ShallowConvNet	Backbone	76.04 ± 1.82	45.56 ± 5.66	85.07 ± 1.68	55.28 ± 2.65	62.29 ± 0.90	51.32 ± 1.71	69.58 ± 5.41	76.25 ± 1.19	74.79 ± 0.53	66.24 ± 1.31
	Proposed	**78.26** **±1.91**	**46.18** **±6.53**	**86.32** **±1.53**	**56.94** **±3.47**	**62.85** **±1.68**	**52.36** **±2.50**	**72.64** **±6.47**	**77.64** **±2.05**	**76.04** **±0.65**	**67.69** ±1.54^**^

It shows average accuracy ± standard deviation for each method, which is based on five repeated experiments and their averaged results. Wilcoxon's signed-rank test was used for statistical significance (Wilcoxon, 1992) (^*^p < 0.05, ^**^p < 0.01). The highest results for each subject, from both the original backbone network and our proposed method, are highlighted in boldface.

Table 3 also indicates that our proposed method helps achieve performance improvement for the backbone networks on the KU-MI dataset. In particular, the improvements for EEGNet, DeepConvNet, and ShallowConvNet are 0.71% (*p* < 0.01), 0.18% (*p* = 0.196), and 2.24% (*p* < 0.01), respectively.

**Table 3:** The performance comparison table presents average accuracy ± standard deviation, based on five random seeds, for different backbone networks with and without the proposed LRP-based feature selection applied to the KU-MI dataset.

**Comparison methods**		**Mean**
EEGNet	Backbone	65.94 ± 0.28
	Proposed	**66.65** ±0.34^**^
DeepConvNet	Backbone	65.73 ± 0.46
	Proposed	**65.91** **±0.37**
ShallowConvNet	Backbone	61.40 ± 0.75
	Proposed	**63.64** ±0.86^**^

A *t*-test was employed to assess statistical significance (Student, 1908) (^*^: *p* < 0.05, ^**^: *p* < 0.01). The highest results for each subject, from both the original backbone network and our proposed method, are highlighted in boldface.

Table 4 demonstrate the performance of our LRP-based feature selection method in comparison to conventional feature selection methods for each backbone network on the BCI Competition IV-2a dataset. The comparison methods include analysis of variance (ANOVA)-based feature selection (Salami et al., 2017; Miah et al., 2020), minimum-redundancy-maximum-relevance (mRMR)-based feature selection (Peng et al., 2005; Jenke et al., 2014; Al-Nafjan, 2022), and recursive feature elimination (RFE)-based feature selection (Cai et al., 2018; Jiang et al., 2020; Al-Nafjan, 2022). The Wilcoxon signed-rank test was applied to assess differences between each competitive method and our LRP-based feature selection (Wilcoxon, 1992). While our LRP-based feature selection method performed comparably to other conventional methods across different backbone networks, statistical analysis showed no significant differences between the methods. More detailed discussions about these results can be found in the discussion section.

**Table 4:** The comparison table illustrates our LRP-based feature selection and traditional machine learning-based feature selection methods for each backbone network on the BCI Competition IV-2a dataset.

**Backbone networks**	**Comparison methods**	**Subjects**									**Mean**	
		**S01**	**S02**	**S03**	**S04**	**S05**	**S06**	**S07**	**S08**	**S09**		
EEGNet	ANOVA-based	78.19 ± 3.4	58.47 ± 1.88	89.58 ± 3.12	66.46 ± 2.91	72.71 ± 2.23	**60.21** **±2.81**	**73.54** **±3.05**	79.51 ± 2.24	**78.68** **±3.38**	**73.04** **±2.78**	
	mRMR-based	78.33 ± 3.12	58.54 ± 2.46	89.72 ± 3.20	66.25 ± 2.60	**72.92** **±2.33**	59.93 ± 3.24	73.40 ± 3.16	**79.58** **±2.37**	78.47 ± 3.80	73.02 ± 2.92	
	RFE-based	**78.61** **±3.66**	58.61 ± 2.53	89.72 ± 2.94	**66.94** **±2.24**	72.57 ± 2.07	60.07 ± 3.50	73.54 ± 3.55	79.30 ± 2.81	78.54 ± 3.36	73.10 ± 2.96	
	LRP-based (proposed)	78.20 ± 3.38	**58.82** **±2.67**	**89.79** **±2.56**	66.18 ± 2.76	72.57 ± 2.70	59.93 ± 3.18	73.19 ± 2.98	78.89 ± 2.78	78.13 ± 4.34	72.86 ± 0.71	
DeepConvNet	ANOVA-based	75.97 ± 1.54	47.50 ± 2.19	**88.40** **±1.94**	68.33 ± 2.25	74.58 ± 1.19	56.94 ± 2.29	74.44 ± 1.14	78.05 ± 2.19	**83.13** **±1.66**	71.93 ± 1.82	
	mRMR-based	76.04 ± 1.63	47.43 ± 2.05	**88.40** **±1.94**	68.47 ± 2.33	74.72 ± 1.29	56.94 ± 2.40	**74.65** **±1.28**	78.05 ± 2.19	83.06 ± 1.86	**71.98** **±1.88**	
	RFE-based	75.97 ± 1.60	47.22 ± 1.92	88.12 ± 1.78	**68.54** **±2.44**	74.65 ± 1.32	57.09 ± 2.32	74.44 ± 1.00	78.06 ± 1.40	82.71 ± 1.65	71.87 ± 1.71	
	LRP-based (proposed)	**76.25** **±1.83**	**47.50** **±1.60**	87.99 ± 2.25	68.40 ± 2.75	**75.28** **±1.13**	**57.22** **±2.94**	74.58 ± 0.90	**78.12** **±1.85**	82.36 ± 1.37	71.97 ± 0.79	
ShallowConvNet	ANOVA-based	77.71 ± 1.28	45.42 ± 6.46	87.57 ± 1.71	55.21 ± 3.17	62.71 ± 2.20	51.74 ± 2.20	72.36 ± 6.22	77.64 ± 1.64	75.49 ± 1.00	67.31 ± 2.88	
	mRMR-based	77.98 ± 1.66	45.49 ± 6.07	**87.85** **±1.49**	54.58 ± 2.79	62.64 ± 1.96	52.15 ± 1.89	72.02 ± 6.41	**77.92** **±1.27**	75.62 ± 0.62	67.36 ± 2.68	
	RFE-based	77.08 ± 1.70	45.14 ± 6.62	86.39 ± 1.64	55.41 ± 2.52	**63.40** **±1.91**	52.01 ± 2.44	72.57 ± 6.74	77.78 ± 1.39	**76.11** **±0.38**	67.32 ± 2.81	
	LRP-based (proposed)	**78.26** **±1.91**	**46.18** **±6.53**	86.32 ± 1.53	**56.94** **±3.47**	62.85 ± 1.68	**52.36** **±2.50**	**72.64** **±6.47**	77.64 ± 2.05	76.04 ± 0.65	**67.69** **±1.54**	

It shows average accuracy ± standard deviation, which is based on five repeated experiments. Wilcoxon's signed-rank test was applied between each competitive method and our LRP-based feature selection (Wilcoxon, 1992). (^*^:*p* < 0.05,^*^*:*p* < 0.01). The highest results for each subject, from both the original backbone network and our proposed method, are highlighted in boldface.

## 4. Discussion

**Figure 3:**
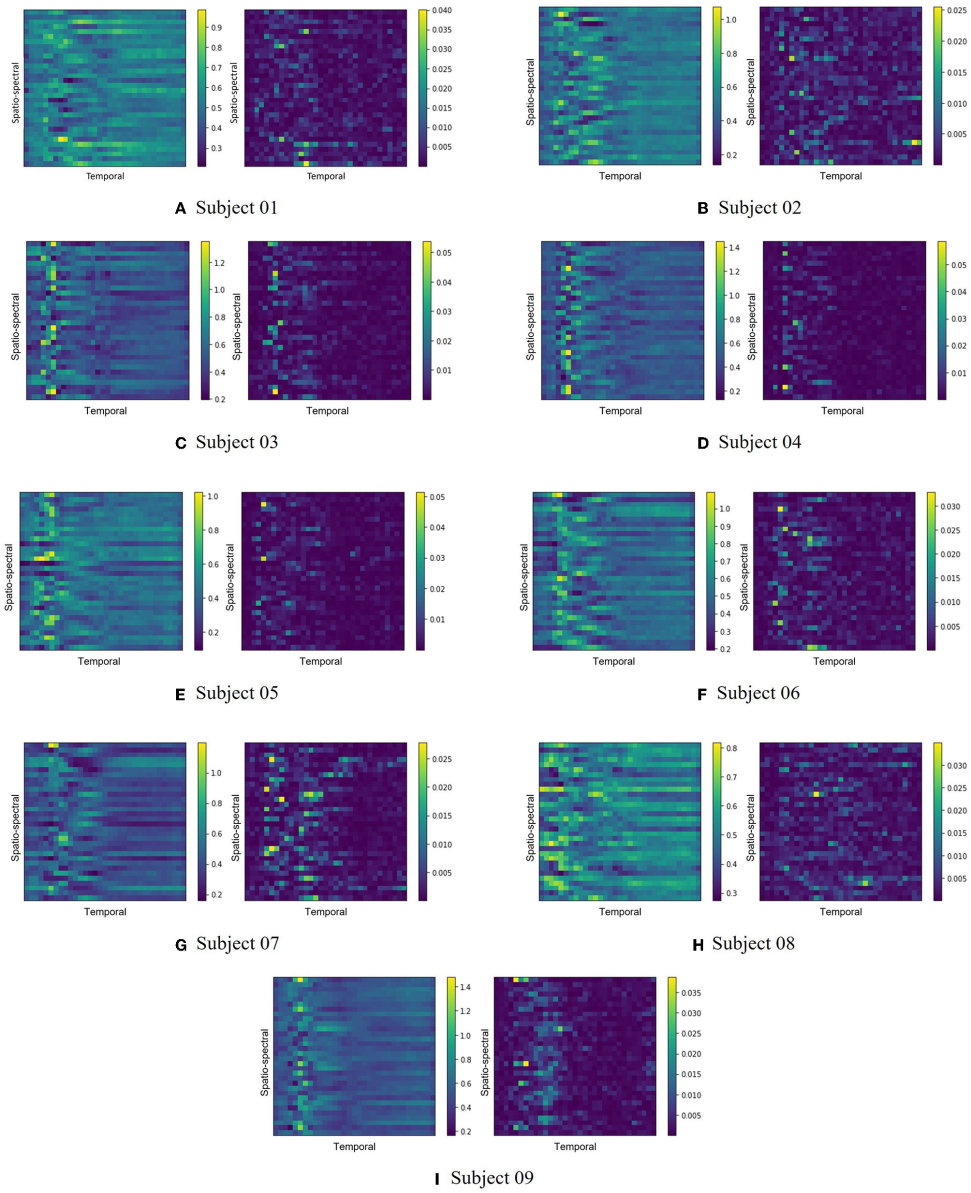
The extracted feature maps and corresponding LRP score heatmaps from the EEGNet backbone network for the BCI Competition IV-2a dataset are depicted. Each subfigure presents the pairs of the average feature map from all trials for each subject (left) and the associated LRP score heatmap (right). Subfigures **(A–I)** respectively represent subjects 1 through 9.

Figure 3 illustrates the feature maps extracted by one of the backbone networks, EEGNet, on the BCI Competition IV-2a dataset. The extracted feature maps, representing the average of all trials for each subject, are shown on the left side of each subfigure, while the corresponding LRP score heatmaps are displayed on the right. According to Figure 3, the patterns of each feature map and their corresponding heatmap differ across subjects. This variation might be related to the dynamics of event-related synchronization (ERS) and event-related desynchronization (ERD) during the imagery tasks as some studies have reported transient desynchronization patterns occurring in the brain and recovering to baseline levels within a specific time frame (Pfurtscheller et al., 1997; Pfurtscheller and Da Silva, 1999; Bartsch et al., 2015). This can emphasize the importance of recognizing subject-specific patterns and conducting appropriate feature selection accordingly.

**Figure 4:**
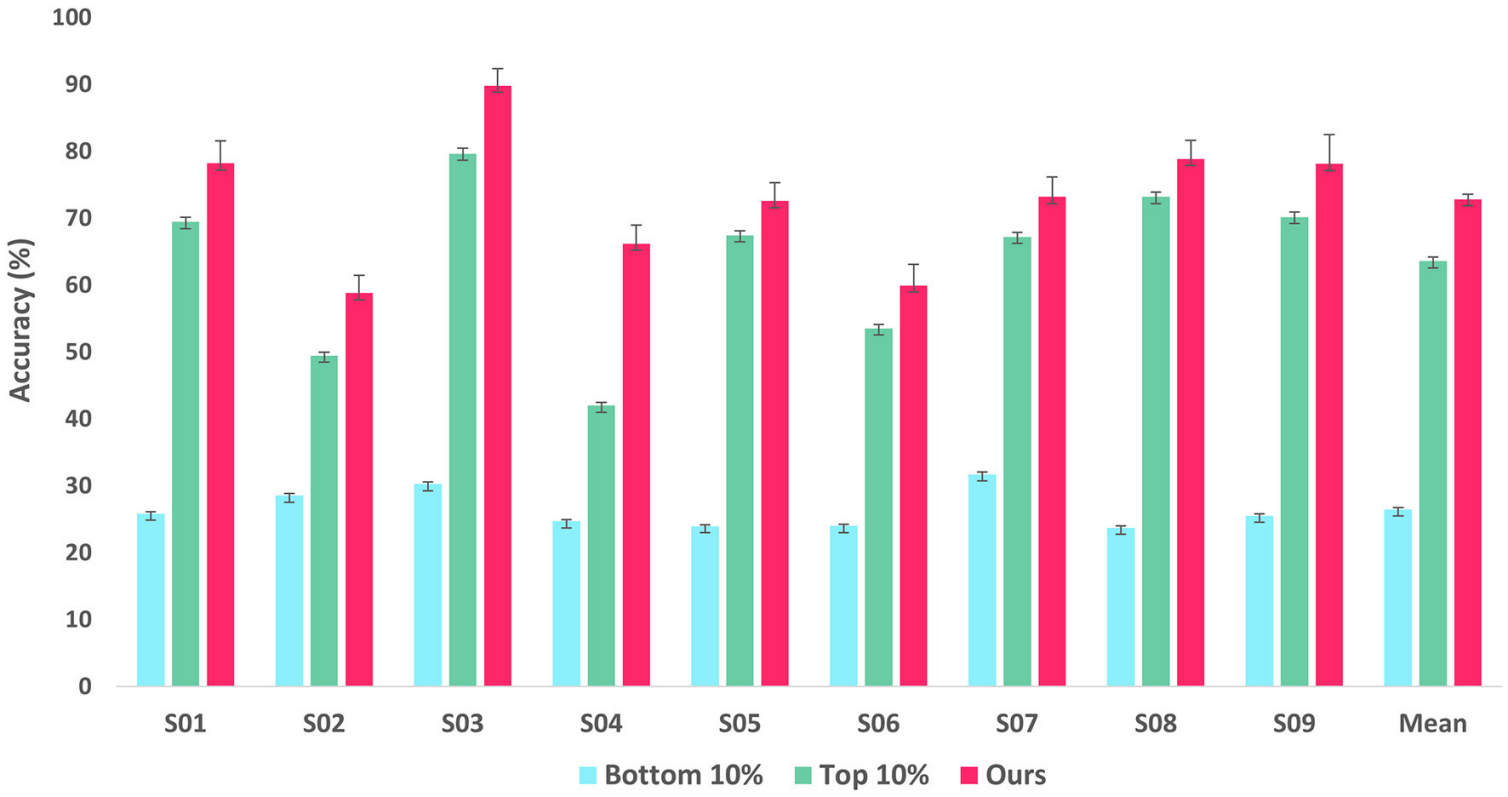
Bar graph showing classification accuracy of EEGNet-based classifiers retrained with two methodologies and our proposed framework on the BCI Competition IV-2a dataset. The first bar represents the classifier retrained with the lowest 10% of features, while the second bar represents the classifier retrained with the highest 10%. The last one represents our proposed framework.

To validate the reliability of LRP-based feature selection, we trained two additional classifiers derived from the EEGNet architecture: one with only the 10% of EEG features with the lowest importance scores, and the other with exclusively the 10% of features with the highest importance scores for training. Of note, the classifier in our model takes the features masked with a position corresponding to the importance scores in the bottom 10%, i.e., it is trained with 90% of features with the highest importance scores. Figure 4 shows the comparative performance of the two classifiers and the classifier in our proposed method in terms of accuracy on the BCI Competition IV-2a dataset. The comparison results show that the model with the 10% lowest features achieved accuracies around 25% close to the chance rate of randomly picking one from four classes over all the subjects while the model with 10% highest features remarkably outperformed the former model for all subjects. Thereby we can assume that the features with the lowest importance scores are mostly class-irrelevant and the features with high importance scores are significantly informative for MI classification. However, more importantly, our model still excels in the two classifiers for all subjects, which can be interpreted that the rest features which have importance scores between the 10% lowest and 10% highest importance scores also contribute to classification for further performance improvement.

**Figure 5:**
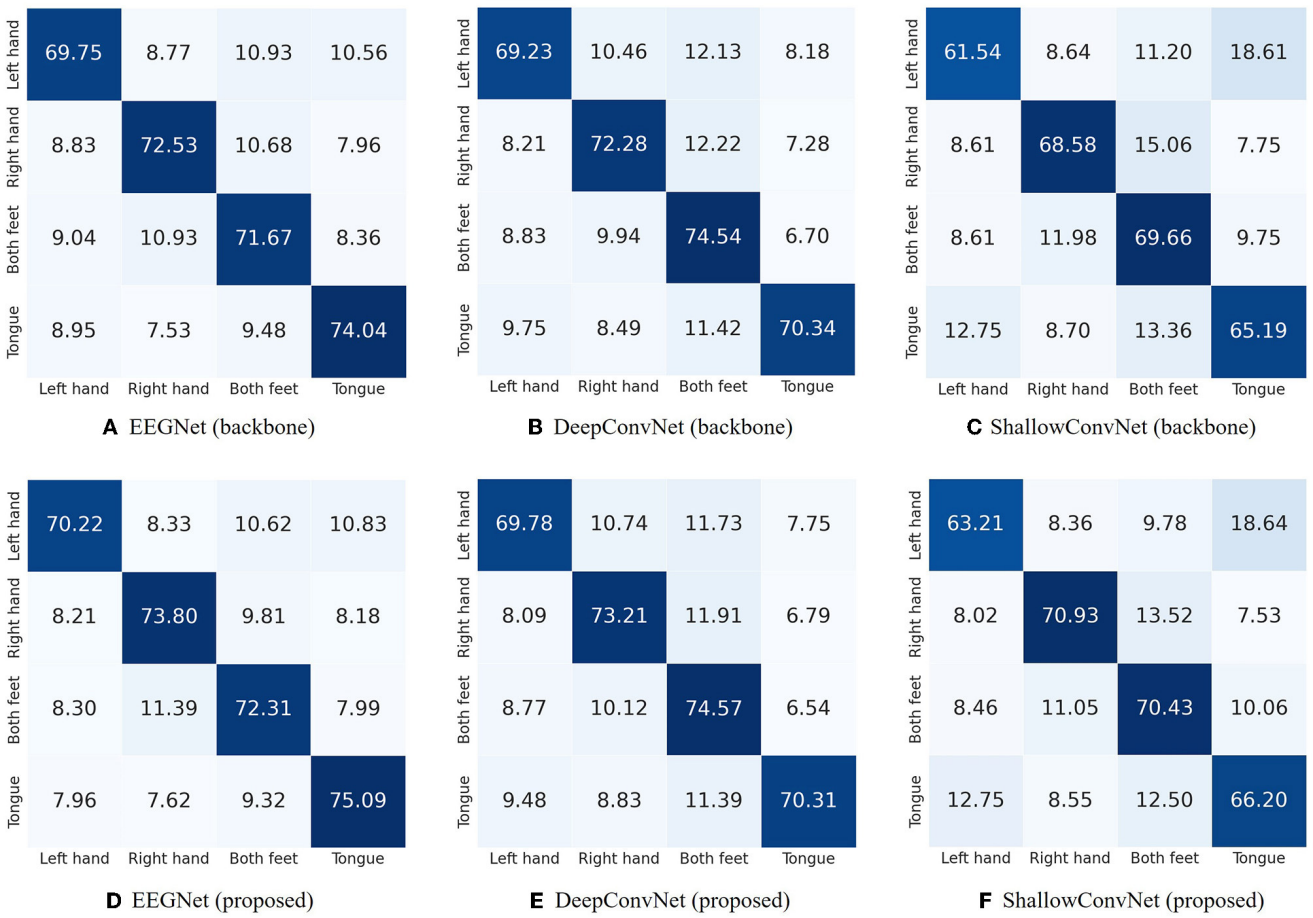
Confusion matrix for EEGNet, DeepConvNet, and ShallowConvNet on the BCI Competition IV-2a dataset. The top row [subfigures **(A–C)**] presents the results for the backbone networks, while the bottom row [subfigures **(D–F)**] shows the outcomes when our LRP-based feature selection is applied to the respective networks.

For a more comprehensive analysis, we evaluated the confusion matrix on each backbone network without and with our proposed feature selection method on the BCI Competition IV-2a dataset, displayed in Figure 5. The results show that our method helps classify most of the MI classes in backbone networks more clearly. For instance, considering ShallowConvNet as one of the three backbone networks, Figures 5C, F demonstrate that our proposed feature selection method improved the performance for each class, with specific improvements in classification outcomes (e.g., Class left hand: 1.67%, Class right hand: 2.35%, Class both feet: 0.77%, and Class tongue: 1.01%). Notably, similar performance improvements were observed across nearly all classes in the other two backbone models as well, highlighting the effectiveness of our proposed method.

Considering the results presented in Table 4, one of the key advantages of our LRP-based feature selection method is that it provides an end-to-end approach, encompassing feature extraction, feature selection, and classification. This is in contrast to traditional conventional methods that often involve separate steps for these tasks. While our LRP-based feature selection method achieved the best performance only when ShallowConvNet was used as a backbone network, the statistical analysis showed that there were no significant differences between our method and the conventional methods. Additionally, the LRP method offers the advantage of allowing for intuitive interpretation of the input feature map, showing which parts contribute to classification. This could potentially facilitate further understanding and refinement of the feature selection process in the BCI fields.

## 5. Limitations and future work

In our study, one of the limitations is that we only investigated the potential of LRP-based feature selection for MI classification. We did not succeed in identifying the optimal proportion of masking that would vary depending on the subject, nor the ideal proportion based on the size of the feature map. This resulted in limited performance improvement compared to the conventional methods. For future work, we could conduct research to determine the optimal proportion of features to be masked while varying the size of the features extracted from the same backbone network architecture. Additionally, exploring how different sizes of feature maps may lead to different results could provide valuable insights, with the ultimate aim to identify the best feature set. Further improvement of our current study also involves propagating the Layer-wise Relevance Propagation (LRP) not only up to the feature map but also to the initial part of the feature extractor. We expect that this will lead to identifying important features from a more diverse range of perspectives, such as temporal, spatial, and spectral domains. Therefore, the subsequent studies will further advance our understanding and the practical application of LRP-based feature selection in the field of MI classification, ultimately resulting in enhanced performance. Drawing from the potential and insights of this study, our research could be also expanded to other BCI paradigms as well.

## 6. Conclusion

In conclusion, we have successfully designed and evaluated the layer-wise relevance propagation (LRP)-based feature selection for class-discriminative EEG features in MI-BCI by examining various backbone networks on two different datasets. The results demonstrated the effectiveness of our proposed LRP-based feature selection across all backbone networks and datasets. Furthermore, to determine the true effectiveness of this approach, we have thoroughly analyzed the LRP-based feature selection using diverse analysis methods, including experiments comparing high-importance scored features and low-importance scored features obtained through LRP, as well as class-specific performance evaluations. Therefore, we claim that the LRP-based feature selection not only demonstrated its effectiveness but also allowed us to identify the most crucial features for classification, as evidenced by our findings. Furthermore, we believe that our LRP-based feature selection approach can potentially be applied to other domains.

## Data availability statement

The original contributions presented in the study are included in the article/supplementary material, further inquiries can be directed to the corresponding author.

## Author contributions

HN and J-MK contributed to the conception and design of the study. WC wrote sections of experiments in the manuscript draft. SB was responsible for creating figures and performing statistical tests. T-EK supervised the project and revised the paper. All authors contributed to manuscript revision, read, and approved the submitted version.
